# Sickness and Vomiting of Pregnancy

**Published:** 1880-11

**Authors:** J. J. Jones


### SICKNESS AND VOMITING OF PREGNANCY.

By DR. J. J. JONES, SR.

This is a malady frequently met with by physicians, and one whose pathology is illy understood and but little considered; yet I know of none other that is calculated to give more embarrassment and anxiety to the practitioner, and none not immediately dangerous to life, that is more distressing to the sufferer; therefore, constituting a strong claim upon us for relief. The ordinary morning sickness of pregnancy I do not propose to consider, as but little if any treatment is required. It leaves behind no ill effects, and usually subsides about the fourth month. But when the sickness and vomiting are persistent, interfering with nutrition, and finally compromising the life of the mother, or child, then the case calls for active interference. The cases that have fallen under my care have usually occurred between the first and fourth month, but quite a number in the latter months of pregnancy, and they have been much more obstinate, and yielded to treatment much less readily than those occurring at the earlier period. This sympathetic vomiting does not usually cause abortion, unless long continued; but the "little squatter-sovereign" seems to occupy quietly the premises, unconscious of the storm that is raging without, and often continues to live even up to the death of the mother, unless dislodged by artificial means—which should always be done as a last resort; many mothers' lives have been saved by this interference.

Notwithstanding the violence and obstinacy of such cases, you seldom find any inflammation of the stomach, or any considerable amount of fever; the sickness and vomiting being entirely reflex, or sympathetic, owing, no doubt, to the intimate connection of the organs of generation with the stomach through the cerebro-spinal, and ganglionic system of nerves. The stomach is almost invariably the first organ to sympathize with the womb, and is usually independent of any noticeable change in temperature or disturbance of the circulation. This sympathy is of various degrees of intensity—sometimes causing a more fastidious taste and appetite, than intense nausea and vomiting. A large majority of women suffer more or less with sickness and vomiting during their pregnancies, yet there are cases that are free from it throughout the whole period. Such cases, it is thought by some writers, are more subject to the diseases incident to childbed than those that have suffered the usual amount of sickness. But why I cannot tell, as such has not been my experience, or, if it is so, it has escaped my observation.

As I remarked at the commencement of this article, the pathology of this affection is illy understood—evidenced by the fact that there is no uniformity of theory or practice among the profession in regard to it. But one authority contends that it is caused by resistance to the normal expansion of the tissues at and immediately surrounding the internal uterine orifice. Therefore, Copeland's method of dilatation must be practiced. Another's, that it is caused by inflammation of the cervix uteri, and, therefore, the cervix should be cauterized with nitrate of silver, or painted with tincture iodine. In fact, theory after theory has been promulgated—leaving its pathology still unsettled; and remedy after remedy put forward as certainly specific; each, in time, to work out its own inefficiency. So we have no certain remedy, or combination of remedies, that we can rely upon in every case, and never can have, while different pathological conditions are found causing the malady in question. The cause must be ascertained in every individual case, and then it will be an easy matter to arrive at the remedy. It has fallen to my lot to treat a great many cases of this affection of every degree of intensity, and the same remedy that has acted like a charm in one case, has utterly failed in another. A few have not been relieved by any of the remedies put forth, and I have been compelled to resort to the induction of premature delivery. Having preserved the notes of a rather extraordinary case which I treated last year, I will give its history :

On the 17th of July, 1879, I was called to see Mrs. B., who resided ten miles from my place of residence. I found her vomiting every half hour, and complaining of intense nausea. She was twenty years of age, and of good constitution, but in a state of extreme emaciation. I was informed that she was in her first pregnancy, then seven months advanced, and that she had been vomiting from fifteen to twenty times daily for the last six weeks, and also that she had suffered more or less from sickness and vomiting during the whole period of her pregnancy. She had been under the treatment of a physician in the neighborhood, but I was unable to ascertain the treatment she had received. I found her so weak and feeble that she was unable to rise up in bed without assistance. Her tongue looked a little red on the edges, but clean, no tenderness over the region of the stomach or bowels on pressure, with temperature at 100 F., and pulse at 108, feeble. Both food and medicines were rejected almost as soon as swallowed. The movement of the child in utero, seemed to excite great nausea, and was frequently the cause of severe retchings. Her urine was examined and found slightly albuminous but normal in quantity. Her bowels were not constipated, but she had passed littte slimy dejections daily. I placed a blister over the stomach, and prescribed subnitrate of bismuth in five grain doses every three hours, and beef-tea enemas every four hours.

July 19th.—No better. I then prescribed oxalate of cerium, and continued the beef tea injections.

July 21st.—Still no improvement. Gave hypodermic injections of morphine, which gave temporary relief, but the vomiting returned in three hours with increased severity; so much so that I was compelled to abandon them, for the time. Prescribed ice, with champagne; continued the beef-tea, etc.

July 23rd.—Unimproved. Dilated the os uteri, and gave injections of chloral with bromide potassium, repeated every four or five hours.

July 25th.—Still vomiting. Dr. Harris, my partner, had accompanied me for the purpose of assisting me in the production of delivery, but we abandoned the idea, at the solicitation of the family, and were also influenced by the fact that the child was still vigorous We then cauterized the neck of the womb with solid nitrate of silver, gave ingluvia, 8 grains every three hours, applied mint poultices to the stomach, and gave beef-tea by enema.

July 27th.—Our patient had spent a miserable night, vomiting almost incessantly. I again insisted on producing delivery, but was again induced to wait by the intercessions of the parents, who did not believe that she could survive the ordeal. I had often relieved intense nausea and vomiting in the non-pregnant with small doses of calomel, frequently repeated, and concluded I would try it in this case. So I left half grain doses of calomel, to be put upon the tongue and swallowed with a little gum-water, every two hours, and not to desist from giving them until the bowels acted freely.

July 29th.—We found our patient this morning relieved, and able to retain light nourishment. She continued to improve under a carefully regulated diet; had no return of vomiting, and went to the period of her pregnancy without further interference.

The above case is interesting on account of the severity and persistency of the symptoms—having resisted all the usual remedies in such cases, and finally succumbed to the alterative influence of mercury. I have often felt that old and tried remedies are too often made to give place to new ones of less potency, and that there is an unnecessary prejudice existing with many physicians in regard to the use of calomel in treating the diseases occurring in pregnancy, that they would give calomel in without hesitation in the non-pregnant.—*Arkansas Medical Monthly.*

